# Attitudes and practices related to hypoglycemia unawareness in patients with type 1 and type 2 diabetes

**DOI:** 10.25122/jml-2024-0005

**Published:** 2024-08

**Authors:** Azam Tarek Alhedhod, Suha Albahrani, Abdullah Almaqhawi, Hussain Sami Alwesaibie, Mohammed Abdulkhaliq Albesher, Jana Mohsen Alwadani, Nawar Ammar Alshakhs, Rawan Mohammed Aldihnayn, Ghadeer Ahmed Al bensaad

**Affiliations:** 1 College of Medicine, King Faisal University, Al Hofuf, Saudi Arabia; 2 Department of Family and Community Medicine, College of Medicine, King Faisal University, Al Hofuf, Saudi Arabia

**Keywords:** attitudes, practices, hypoglycemia, diabetes mellitus

## Abstract

Diabetes is considered one of the most prevalent endocrine metabolic diseases. Monitoring hypoglycemia unawareness is an important component of routine diabetes care and can identify patients at increased risk of a severe hypoglycaemic event. This study aimed to evaluate the frequency of hypoglycemia unawareness and identify the factors contributing to its occurrence. A sample of 390 patients diagnosed with type 1 and type 2 diabetes was interviewed in an endocrine and diabetes center in Al-Ahsa city. Sociodemographic data, risk factors, and Clarke scores were used to evaluate the impairment of hypoglycemia awareness. Reduced awareness of hypoglycemia was found in 93 patients (23.8%). There were no statistically significant differences in the age of the patients, mean age of diagnosis, or cumulative glucose level between patients with awareness and those with reduced awareness (*P* > 0.05). Patients with type 2 diabetes mellitus (T2DM) showed significantly reduced awareness compared to type 1 diabetes (T1DM) (*P* = 0.038). Additionally, there were no statistically significant differences in hypoglycemia awareness between patients who underwent nephropathy screening and those who did not (*P* = 0.523). In conclusion, our study revealed reduced hypoglycemia awareness in 93 patients. However, there was no statistically significant difference related to various factors, including age and cumulative glucose levels. Patients with T2DM showed significantly lower hypoglycaemic awareness compared to patients with T1DM. Further research is needed to evaluate other factors of hypoglycemia unawareness.

## INTRODUCTION

One of the most prevalent endocrine metabolic illnesses presently affecting children and adolescents is type 1 diabetes mellitus (T1DM). According to the World Health Organization (WHO), Saudi Arabia has the second-highest rate of diabetes in the Middle East and is ranked seventh worldwide [1]. T1DM is commonly associated with significant acute and chronic consequences [2]. The hyperglycemia associated with this disease promotes a pathologic attribute involving the vasculature, resulting in both microvascular and macrovascular complications [3]. Diabetic ketoacidosis and severe hypoglycemia are the main life-threatening complications of T1DM that present acutely [4]. Acute hypoglycemia is a consequence of diabetes treatment and is the most prevalent deadly acute complication [5].

As T1DM is insulin-dependent, hypoglycemia is a more pervasive problem for these patients; therefore, patients with T1DM are more prone to the consequences of hypoglycemia [6]. Type 2 diabetes mellitus (T2DM), which is the most common type, develops much later in life than T1DM. T2DM is also referred to as non-insulin dependent because its main treatment is tablets rather than only insulin [7]. Patients with T2DM can control the amount of glucose they make by exercising more, eating fewer high-carbohydrate meals, and losing weight. However, insulin resistance might persist; therefore, patients should continue and adjust as necessary through specialized training, physical activity, food management, body weight management, and the use of medication [8].

Diabetes mellitus is commonly associated with long-term complications, particularly microvascular manifestations such as retinopathy, neuropathy, and nephropathy. Additionally, it can negatively impact cognitive function and various organs, including the heart. Macrovascular consequences of T1DM include atherosclerosis and thrombosis in the heart, peripheral arteries, and brain [9]. Hypoglycaemia is commonly defined as a plasma glucose concentration below 70 mg/dL, but symptoms may not manifest until the concentration falls below 55 mg/dL [10]. In individuals with T1DM, hypoglycemia is a major obstacle to achieving optimal metabolic control and is the most prevalent acute complication [11]. Intensive glycaemic control attempts increase the risk of hypoglycemia: patients with T1DM who rely on insulin are at a high risk of experiencing severe hypoglycemia, which is marked by blood glucose levels dipping below 50 mg/dL. This vulnerability remains consistent across all treatment regimens [12]. Regarding the impact of hypoglycemia, one study found a correlation between hypoglycemia and cognitive impairment in older patients with type 2 diabetes [13]. According to Whitmer *et al*., elderly patients with type 2 diabetes who experience recurrent severe hypoglycemia are at a statistically significant risk of developing dementia [14]. Individuals with severe hypoglycemia can experience a six-fold increase in the risk of diabetes-related fatality compared to patients who do not have severe hypoglycemia. Experiencing frequent hypoglycaemic episodes can damage the counter-regulatory system and increase the risk of developing hypoglycemia unawareness (HU) [15].

Usually, in the early stages of hypoglycemia, the autonomic nervous system is activated, causing a variety of symptoms that can alert patients and allow them to identify their hypoglycemia and mitigate it. Hypoglycemia can occur in the absence of these warning symptoms in a phenomenon called HU, which occurs when neuroglycopenia begins before the development of autonomic warning signals. HU is a substantial impediment to well-controlled diabetes and a better quality of life. About 40% of people with T1DM, and a lower percentage of those with T2DM, experience HU. Patients with HU may, however, show symptoms of neuroglycopenia in more severe hypoglycemia caused by glucose deprivation of the central nervous system [16,17]. Despite the lack of understanding of the exact mechanisms involved in HU, it is evident that typical compensatory processes, such as glucagon secretion and epinephrine release, fail to initiate. As a result, it becomes necessary to intervene in order to normalize blood glucose levels [18]. The objective of this study was to evaluate the frequency of HU and identify the factors that contribute to its occurrence.

## MATERIAL AND METHODS

### Study design

This cross-sectional study involved patients diagnosed with T1DM and T2DM who were on insulin treatment. Participants were recruited from the Al-Ahas Governorate of Saudi Arabia to assess demographic variables, HU, and factors associated with HU. Convenience sampling was employed to select subjects from the Endocrine and Diabetes Centre in Al-Ahsa. The research was conducted between April–July 2023.

### Participants

We recruited participants from the Endocrine and Diabetes Center in Al-Ahsa using a convenience sampling method. A total of 390 young adults were recruited to participate in this study voluntarily. The mean age of patients was 44.6 ± 17.7 years. The sample size was determined by considering the values derived from a similar previous study by Elshebiny *et al*., in which a sample mean of 4.51 was found [19]. A minimum sample of 453 was determined to be acceptable for our study. The recommended sample size was calculated using nMaster 2.0 (CMC, Vellore, India). The inclusion criteria for the study encompassed individuals of all ages diagnosed with either type 1 or type 2 diabetes who were Saudi citizens residing in Al-Ahsa. Exclusion criteria included non-Saudi citizens and individuals without a diabetes diagnosis.

### Data collection

Demographic variables, HU, and factors associated with HU were evaluated using a questionnaire administered through face-to-face interviews. The questionnaire's validity was assessed by three independent experts, who confirmed its suitability for the study. The validity of the questionnaire was assessed by three experts and found to be suitable. The study adhered to the Strengthening the Reporting of Observational Studies in Epidemiology (STROBE) guidelines, ensuring rigorous and transparent reporting of observational research.

### Knowledge of hypoglycemia symptoms assessment

To achieve the study objectives, demographic information was collected, including age, gender, type of diabetes, age at diabetes onset, duration of diabetes, HbA1c levels, eye and kidney screening results, and insulin regimen. Several questions were asked to assess hypoglycemia and its associated unawareness.

A series of questions were designed to evaluate participants' experiences with hypoglycemia, focusing on the following aspects:

- The frequency of hypoglycemia symptoms when blood glucose levels are low.
- Whether any previously experienced hypoglycemia symptoms have diminished over time.
- The frequency of severe hypoglycaemic episodes, defined by a blood glucose level of less than 53 mg/dL, in the preceding six months.
- The frequency of severe hypoglycaemic episodes associated with seizures, loss of consciousness, or requiring intravenous glucose.
- The frequency of experiencing hypoglycemia symptoms when blood glucose levels are below 70 mg/dL.
- The frequency of not experiencing hypoglycemia symptoms despite blood glucose levels falling below 70 mg/dL.
- The threshold blood glucose level at which the patient begins to experience hypoglycemia symptoms.

Hypoglycemia awareness status was determined by the Clarke score questionnaire, which consists of eight questions that evaluate how patients perceive and experience hypoglycaemic events. Responses to these questions generated a score ranging from 0 to 7. A score of 4 or higher indicates impaired hypoglycemia (IAH) awareness, while a score of 2 or lower suggests normal awareness. A score of 3 indicates an undetermined awareness status [20].

### Statistical analysis

Data were downloaded and entered into Microsoft Excel by a trained investigator, and statistical analysis was conducted using SPSS v. 23 (IBM Corp., USA) by an independent biostatistician. Continuous variables were expressed as mean and standard deviation and/or median and interquartile range after testing them for normality using the Shapiro–Wilk test. Categorical variables were summarized as proportions and frequencies. Any possible relationships among the variables were analyzed using Pearson’s chi-square test. A factorial ANOVA model was used to assess the relationships between the independent and dependent variables (attitude and practice scores). A *P* value ≤ 0.05 was considered statistically significant, with a 95% confidence interval and 20% β-error.

## RESULTS

The analysis included data from 390 patients with diabetes, of whom 51.6% were men and 48.4% were women. The mean age of the patients was 44.6 ± 17.7 years, and the mean age at diagnosis of diabetes was 30.4 ± 16.6 years. The cumulative HbA1c level was 9.70 ± 17.81. A total of 344 patients (88.2%) underwent a retinal examination, with 219 (63.7%) doing so annually. Type 1 diabetes mellitus (T1DM) was present in 41.3% of participants, while 58.7% had type 2 diabetes mellitus (T2DM). Most patients (98.5%) used insulin injections, and 79.7% underwent nephropathy screening (Table 1).

**Table 1:** Patient demographics and diabetes characteristics

		*n*	%
Gender	Female	189	48.5
	Male	201	51.5
Age of patients (mean)		44.6 ±17.7 years	
Age of diagnosis (mean)		30.4 ± 16.6 years	
Age of diagnosis	1-5 years	62	15.9
	5-10 years	98	25.1
	10-15 years	64	16.4
	15-20 years	55	14.1
	>20 years	111	28.5
Cumulative glucose (HbA1c)		9.70 ± 17.81	
Retinal examination	No	46	11.8
	Yes	344	88.2
Frequency of retinal examination (n = 344)	Annually	219	63.7
	Not annually	125	36.3
Type of insulin used	Insulin injection	384	98.5
	Insulin pump	6	1.5
Type of diabetes	Type 1	161	41.3
	Type 2	229	58.7
Nephropathy screening	No	79	20.3
	Yes	311	79.7
Hypoglycemia unawareness	No	297	76.2
	Yes	93	23.8

Reduced awareness of hypoglycemia was observed in 93 patients (23.8%). There were no statistically significant differences in age, age at diagnosis, or cumulative glucose levels between patients with normal awareness and those with reduced awareness (*P* > 0.05). No statistically significant differences were observed in hypoglycemia awareness between patients who underwent a retinal examination and those who did not (*P* = 0.274). Additionally, no differences were observed in hypoglycemia awareness between patients using different types of insulin (*P* = 0.130). However, patients with T2DM showed significantly reduced awareness compared to patients with T1DM (*P* = 0.038). Furthermore, there were no statistically significant differences in hypoglycemia awareness between patients who underwent nephropathy screening and those who did not (*P* = 0.523) (Table 2).

**Table 2:** Comparison of hypoglycemia awareness between various patient characteristics

Aware		Hypoglycemia awareness		*P* value
		Reduced awareness		
Age (years)		44.7 ± 17.1	44.1 ± 19.7	0.777
Mean age of diagnosis (years)		30.8 ± 15.9	29.2 ± 18.6	0.404
Cumulative glucose (HbA1c)		9.47 ± 17.08	10.44 ± 20.05	0.651
Diagnosed with diabetes	1-5 years	48 (77.4%)	14 (22.6%)	0.913
	5-10 years	75 (76.5%)	23 (23.5%)	
	10-15 years	47 (73.4%)	17 (26.6%)	
	15-20 years	40 (72.7%)	15 (27.3%)	
	>20 years	87 (78.4%)	24 (21.6%)	
Retinal examination	No	3(82.6%)	8 (17.4%)	0.274
	Yes	259 (75.3%)	85 (24.7%)	
Frequency of retinal examination	Annually	170 (77.6%)	49 (22.4%)	0.184
	Not annually	89 (71.2%)	36 (28.8%)	
Type of insulin used	Insulin injection	294 (76.6%)	90 (23.4%)	0.130
	Insulin pump	3 (50.0%)	3 (50.0%)	
Type of diabetes	Type 1	114 (70.8%)	47 (29.2%)	0.038*
	Type 2	183 (79.9%)	46 (20.1%)	
Nephropathy screening	No	58 (73.4%)	21 (26.6%)	0.523
	Yes	239 (76.8%)	72 (23.2%)	

* P < 0.05 (significant)

Clarke scores were analyzed across various patient characteristics. No statistically significant differences in Clarke scores were found based on the time since diabetes diagnosis (*P* = 0.580) or gender (*P* = 0.091). However, patients with T1DM had significantly higher mean Clarke scores (9.80 ± 1.3) compared to those with T2DM (9.42 ± 1.3; *P* = 0.005) (Table 3).

**Table 3:** Comparison of Clarke scores in patients with various characteristics

		n	Mean	SD	*P* value
Time since diagnosis	1–5 years	62	9.60	1.3	0.580
	10–15 years	64	9.70	1.4	
	15–20 years	55	9.73	1.5	
	5–10 years	98	9.59	1.2	
	>20 years	111	9.41	1.3	
Gender	Female	189	9.46	1.2	0.091
	Male	201	9.69	1.4	
Type of diabetes	Type 1	161	9.80	1.3	0.005
	Type 2	229	9.42	1.3	

* P < 0.05 (significant) 95% confidence interval

## DISCUSSION

The analysis of data from 390 patients with diabetes yielded several noteworthy insights. The nearly equal gender distribution (51.6% men, 48.5% women) suggests that the study captured a representative sample. The mean age of 44.6 years indicates that diabetes affects a broad age range, highlighting the need for both early intervention and ongoing management across different life stages. The mean age of diabetes diagnosis at 30.4 years suggests that diabetes is being diagnosed relatively early in adulthood, which underscores the importance of early screening and preventive measures. The reported mean cumulative glucose level (HbA1c) of 9.70 ± 17.81, although variable, suggests that glycaemic control could be further optimized in this population.

Our study’s observation of a high retinal examination rate (88.2%) underscores a positive shift in patient engagement with recommended screenings for diabetic complications. This outcome is consistent with trends observed in prior research. For example, a study by Hatef *et al*. [21] demonstrated a substantial improvement in the completion of annual diabetic eye examinations, with rates rising from 47.89% in 2010 to 78.07% in 2012 (*P* < 0.001), further emphasizing the progress made in enhancing patient involvement in screening efforts. Among those who underwent a retinal examination, 63.7% did so annually, indicating consistent adherence to medical recommendations.

The distribution of T1DM (41.3%) and T2DM (58.7%) among patients highlights the study’s relevance to both diabetic populations. The prevalence of insulin injections as the primary treatment method (98.5%) observed in our study underscores the pivotal role of insulin therapy in effectively managing diabetes in our study population. This finding aligns with and further substantiates the trends identified in previous studies. For instance, Baruah *et al*. [22] reported that 66.08% of their study population utilized pen devices for insulin administration, while 31.76% used insulin syringes, highlighting the widespread adoption of insulin-based treatments. Moreover, concerns about the lifelong nature of insulin treatment, physical apprehensions about injections, misconceptions surrounding insulin, and fears of diabetes-related complications were highlighted by Liu *et al*. [23], shedding light on the multifaceted issues surrounding the insulin therapy that our study found to be so prevalent. Furthermore, the observation that 79.7% of patients engaged in nephropathy screening suggests a general awareness of the importance of monitoring for kidney-related complications, which is vital in the long-term management of diabetes.

Our examination revealed that 23.8% of patients demonstrated reduced awareness of hypoglycemia symptoms, consistent with previous research on impaired awareness of hypoglycemia (IAH). IAH is recognized as a syndrome in which individuals experience a diminished or no ability to detect warning symptoms of hypoglycemia, a phenomenon elucidated by Stefenon *et al*. [24]. Correspondingly, Plourde *et al*. [25] emphasized that patients with IAH lose their capacity to perceive the onset of hypoglycemic episodes, which parallels the reduced awareness observed in our study sample. This congruence underscores the importance of our findings in contributing to a broader understanding of hypoglycemia awareness among patients with T1DM and aligns with the established notions surrounding IAH in the literature.

Sociodemographic data, such as patient age, age of diabetes diagnosis, and cumulative glucose levels, may not directly influence hypoglycemia awareness. This aligns with the studies of Almigbal and AlTwoayan *et al*., which reported no significant association between various demographic and health-related factors and hypoglycemia awareness [26,27]. These studies collectively suggest that factors like age, education, occupation, and chronic illness history may not play a significant role in predicting hypoglycemia awareness.

There was no significant difference in hypoglycemia awareness between individuals who underwent a retinal examination and those who did not. This suggests that retinal health may not strongly correlate with awareness of hypoglycemia. This finding contrasts with the possibility raised by Khan *et al*. [28], who suggested that individuals with retinal disease might have greater sensitivity to hypoglycemia. Additionally, Singh *et al*. [29] reported that a significant percentage of study participants were aware of the link between diabetes and eye damage, including the risk of blindness. These divergent findings highlight the complexity of the relationships between retinal health, awareness of hypoglycemia, and diabetes-related complications.

The lack of variation in hypoglycemia awareness between patients who used different types of insulin suggests that the choice of insulin treatment may not be a decisive factor in predicting awareness levels. This finding aligns with previous studies that observed limited hypoglycemia awareness variability based on insulin type [30,31].

However, the finding that patients with T2DM had significantly reduced awareness compared to those with T1DM is notable. This could be attributed to differences in disease progression, medication regimens, or underlying physiological differences between the two types of diabetes. This finding aligns with Meijl *et al*. [32], who reported reduced hypoglycemia awareness in T2DM patients. Additionally, the absence of significant differences in hypoglycemia awareness between patients who underwent nephropathy screening and those who did not indicate that nephropathy screening adherence might not directly affect hypoglycemia awareness levels. This is consistent with conclusions from previous investigations [33,34].

The findings must be interpreted in light of several limitations. The focus on a specific geographic region, Al-Ahsa, Saudi Arabia, limits the generalizability of the results to broader populations. Its cross-sectional design prevents the establishment of causal relationships and temporal sequences, limiting the ability to draw definitive conclusions about the relationship between hypoglycemia awareness and its associated factors. Reliance on self-reported data, particularly for variables like retinal examination and nephropathy screening, introduces the potential for recall bias, which might affect the accuracy of these measurements. Gathering data at a single point might not fully capture the dynamic nature of hypoglycemia awareness.

## CONCLUSION

Our study identified reduced hypoglycemia awareness in 93 patients, with no statistically significant differences observed across various factors, including age and cumulative glucose levels. Notably, patients with T2DM exhibited significantly lower hypoglycemia awareness compared to those with T1DM. Further research is needed to evaluate other factors of hypoglycemia unawareness. We recommend using multiple assessment tools, such as the Clarke, Gold, and Pedersen-Bjergaard scoring systems, for a more accurate evaluation of impaired hypoglycemia awareness, as relying on a single system may not provide sufficient accuracy. Additionally, considering other relevant factors—including the use of oral antidiabetic medications, mecobalamin supplementation, smoking habits, continuous glucose monitoring, marital status, and the presence of diabetic peripheral neuropathy—could enhance the evaluation and management of hypoglycemia unawareness in patients with diabetes.

## Personal thanks

We would like to express our gratitude to the participants for their time and passion during the research, without which this project would not have been possible.
